# Improved dye-sensitized solar cell with a ZnO nanotree photoanode by hydrothermal method

**DOI:** 10.1186/1556-276X-9-206

**Published:** 2014-05-02

**Authors:** Shou-Yi Kuo, Jui-Fu Yang, Fang-I Lai

**Affiliations:** 1 Department of Electronic Engineering, Chang Gung University, 259 Wen-Hwa 1st Road, Kwei-Shan, Tao-Yuan 333, Taiwan; 2 Department of Photonics Engineering, Yuan-Ze University, 135 Yuan-Tung Road, Chung-Li 32003, Taiwan; 3 Advanced Optoelectronic Technology Center, National Cheng-Kung University, Tainan 701, Taiwan

**Keywords:** Zinc oxide, Dye-sensitized solar cell, Nanorods, Tree-like

## Abstract

This study investigated the influence of ZnO nanostructures on dye adsorption to increase the photovoltaic conversion efficiency of solar cells. ZnO nanostructures were grown in both tree-like and nanorod (NR) arrays on an AZO/FTO film structure by using a hydrothermal method. The results were observed in detail using X-ray diffraction, field-emission scanning electron microscopy (FE-SEM), UV-visible spectrophotometry, electrochemical impedance spectroscopy, and solar simulation. The selective growth of tree-like ZnO was found to exhibit higher dye adsorption loading and conversion efficiency than ZnO NRs. The multiple ‘branches’ of ‘tree-like nanostructures’ increases the surface area for higher light harvesting and dye loading while reducing charge recombination. These improvements result in a 15% enhancement in power conversion. The objective of this study is to facilitate the development of a ZnO-based dye-sensitized solar cell.

## Background

Dye-sensitized solar cells (DSSCs) have attracted much attention as the next-generation solar cell. DSSCs have been widely researched because of their low cost and high energy conversion efficiency. In a functioning DSSC, photoexcited electrons in the sensitizer are injected into the conduction band of a semiconductor. A charge mediator, i.e., a proper redox couple, must be added to the electrolyte to reduce the oxidized dye. The mediator must also be renewed in the counter electrode, making the photoelectron chemical cell regenerative [1]. At present, the photoelectrochemical system of DSSC solar cells incorporates a porous-structured wide band gap oxide semiconductor film, typically composed of TiO_2_ or ZnO.

The single-cell efficiency of 12.3% has persisted for nearly two decades [2]. This conversion efficiency has been limited by energy damage that occurs during charge transport processes. Specifically, electrons recombine with either oxidized dye molecules or electron-accepting species in the electrolyte [3-5]. This recombination problem is even worse in TiO_2_ nanocrystals because of the lack of a depletion layer on the TiO_2_ nanocrystallite surface, which becomes more serious as the photoelectrode film thickness increases [6].

In response to this issue, this study suggests ZnO-based DSSC technology as a replacement for TiO_2_ in solar cells. Like TiO_2_, ZnO is a wide band gap (approximately 3.3 eV at 298 K) semiconductor with a wurtzite crystal structure. Moreover, its electron mobility is higher than that of TiO_2_ for 2 to 3 orders of magnitude [7]. Thus, ZnO is expected to show faster electron transport as well as a decrease in recombination loss.

However, reports show that the overall efficiency of TiO_2_ DSSCs is far higher than that of ZnO. The highest reported efficiency of 5.2% for ZnO DSSCs is surpassed by 6.3% efficiency for TiO_2_ thin passivation shell layers [7]. The main problem is centered on the dye adsorption process in ZnO DSSCs. The high acidity of carboxylic acid binding groups in the dyes can lead to the dissolution of ZnO and precipitation of dye-Zn^2+^ complexes. This results in a poor overall electron injection efficiency of the dye [8-10].

There are multiple approaches for increasing the efficiency of ZnO DSSCs. The introduction of a surface passivation layer to a mesoporous ZnO framework is one possibility, but it may complicate dye adsorption issues. Alternatively, the internal surface area and morphology of the photoanode could be changed to replace the conventional particulate structures. However, the diffusion length and the surface area are incompatible with one another. Increasing the thickness of the photoanode allows more dye molecules to be anchored, but electron recombination becomes more likely because of the extended distance through which electrons diffuse to the TCO collector. Therefore, the structure of the charge-transporting layer should be optimized to achieve maximum efficiency while minimizing charge recombination.

The insufficient surface is as of 1D nanostructures limit the performance of DSSCs to a relatively low level. Accordingly, a photoanode with a highly branched network could yield greater photoconversion efficiency than 1D nanostructures because dye loading can be enriched without sacrificing electron transport properties [10]. In addition, the highly branched tree-shaped structure possesses larger pores, creating a better transport route for electrolyte diffusion. Researchers have studied many 1D nanostructures, namely, nanowires [11-14], nanoflowers [15], nanotubes [11,16], nanosheets [17,18], nanobelts [11,16], and nanotips [19]. These nanostructures are expected to significantly ameliorate the electron diffusion length in photoelectrode films. By providing a direct conduction pathway for the fast collection of photogenerated electrons, they decrease the potentiality of charge recombination during interparticle percolation by replacing random polycrystalline TiO_2_ nanoparticle networks with ordered crystalline ZnO semiconductor nanowires (NWs). In the past studies, ZnO nanostructures were typically grown by chemical bath deposition (CBD) [20,21]. This paper presents a discussion on the different surface characterizations of ZnO nanostructures using X-ray diffraction (XRD), field-emission scanning electron microscopy (FE-SEM), UV-visible spectrophotometry, electrochemical impedance spectroscopy (EIS), and solar simulation.

## Methods

**Figure 1:**
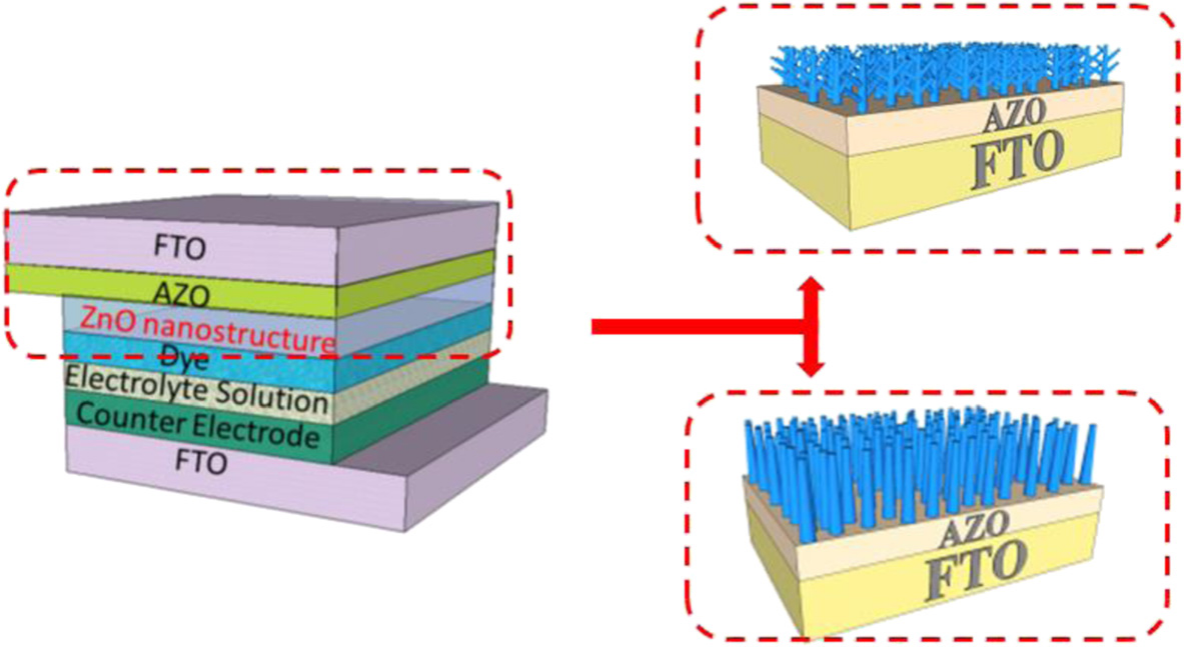
**Schematic illustration of DSSC structures.** The schematic illustration of DSSCs with ZnO nanorods and nanotrees.

In this study, the schematic structures of DSSCs with ZnO nanorods and nanotrees are shown in Figure 1. First, using RF sputtering, an Al-doped ZnO (AZO) seed layer (approximately 300 nm) was deposited on a fluorine-doped SnO_2_ (FTO)-coated glass with a sheet resistance of 8 Ω/sq. The scope of the seed layer definition area was 1 cm^2^ on FTO substrates. These substrates were used for the growth of ZnO nanorods (NRs). The ZnO nanorods were deposited using zinc nitrate (Zn(NO_3_)_2_ · 6H_2_O) and hexamethylenetetramine (HMTA). Both mixtures were dissolved in deionized water to a concentration of 0.02 M and kept under 90°C for 9 h. After the reaction was complete, the resulting ZnO NRs were rinsed with deionized water to remove residual polymer. The NRs with an AZO film were then coated by RF sputtering, and the growth process was repeated to create tree-like ZnO structures from the nanorods.

D-719 dye, *cis*-bis(isothiocyanato)bis(2,2′-bipyridyl-4,4′-dicarboxylato)ruthenium(II)bis-tetrabutylammonium (Everlight Chemical Industrial Corp., Taipei, Taiwan), was dissolved in acetonitrile for the preparation of the 0.5 mM dye solution. Dye sensitization was conducted by soaking the ZnO photoelectrodes in D-719 dye at room temperature for 2 h. A sandwich-type configuration was employed to measure the presentation of the DSSCs. An active area of 1 cm^2^ was assembled by using a Pt-coated FTO substrate as a counter electrode and the Pt/FTO was heated at 200°C for 30 min in air. The DSSC cell was sealed using the polymer resin to act as a spacer. The electrolyte was injected into the space between the electrodes from these two holes, and then these two holes were sealed completely by Surlyn (DuPont, Taipei, Taiwan).

## Results and discussion

**Figure 2:**
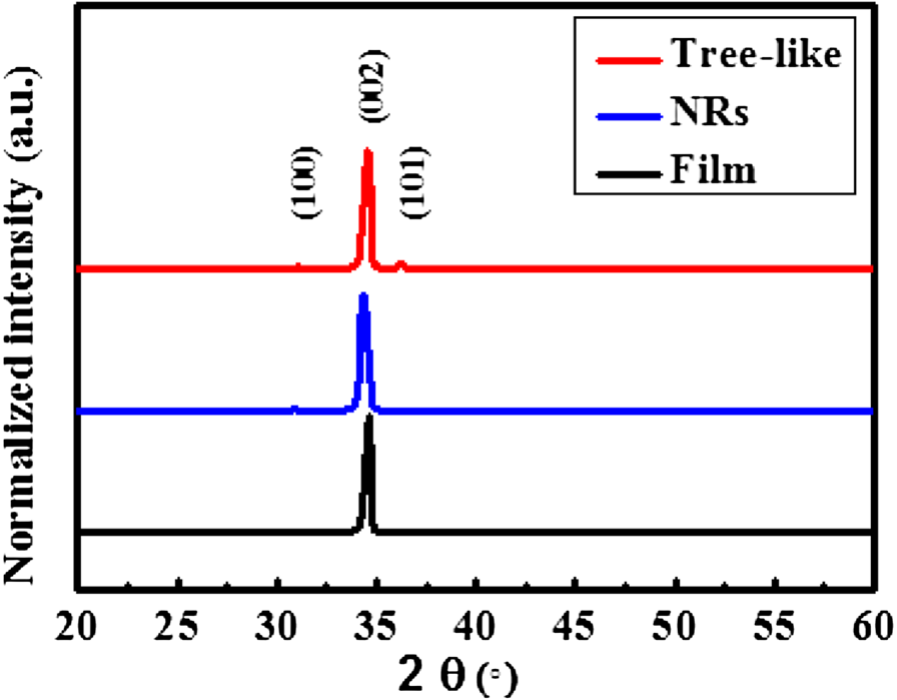
**XRD patterns.** The XRD patterns of different ZnO nanostructures.

In this study, high-density long-branched tree-like ZnO structures and NRs were grown on AZO/FTO substrates of photoanodes to increase the optical absorption of the dye. Figure 2 shows the XRD patterns for the AZO thin film, ZnO nanorods, and tree-like ZnO nanostructures, respectively. The crystalline structure was analyzed using XRD measurements according to a *θ*/2*θ* configuration. According to the XRD database, all of the diffraction peaks can be indexed to the hexagonal wurtzite phase of ZnO. In principle, the XRD spectra show that the ZnO films developed without the presence of secondary phases and groups. No Al_2_O_3_ phase was found. Moreover, the much higher relative intensity of the (002) diffraction peak provides evidence that the nanorods are preferentially oriented in the *c*-axis direction perpendicular to the substrate. No other ZnO phase was found. Regarding tree-like ZnO nanostructures, the presence of secondary phases and groups was observed. These secondary phases and groups result from the thin AZO film coating on the ZnO NRs, which served as a seed layer for the tree-like nanostructures.

**Figure 3:**
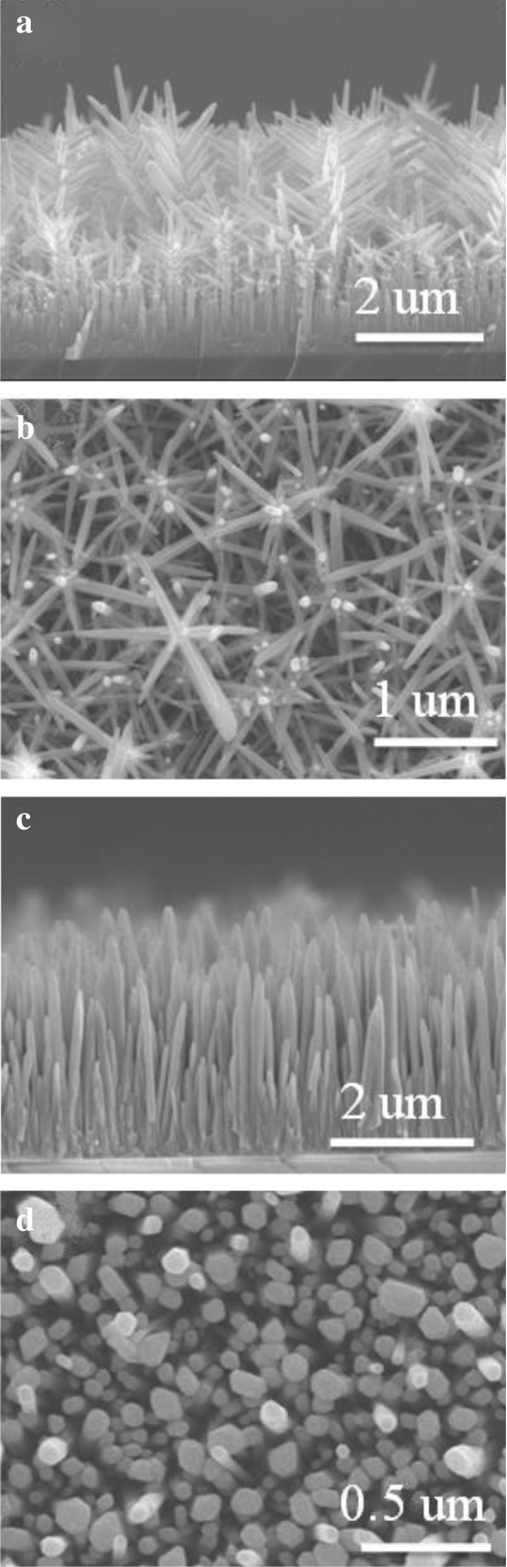
**Scanning electron microscopy images.** SEM images of different ZnO nanostructures on FTO substrates. Side-view **(a,c)** and top-view **(b,d)** of vertically grown tree-like structures.

**Figure 4:**
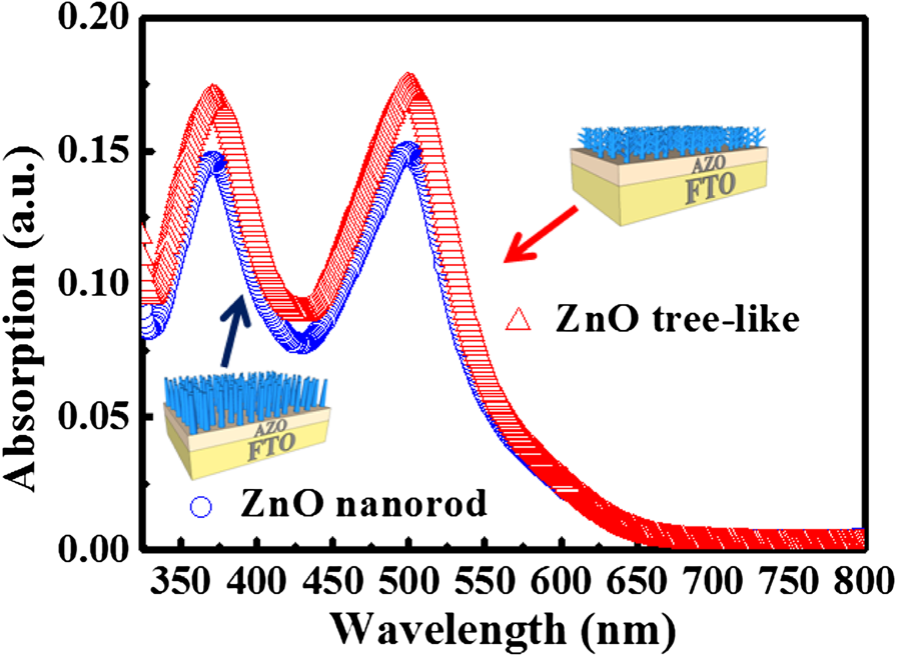
**Absorption spectra of DSSCs with ZnO nanostructures.** Optical absorption spectra of D-719 dye-sensitized ZnO nanostructured electrodes.

**Figure 5:**
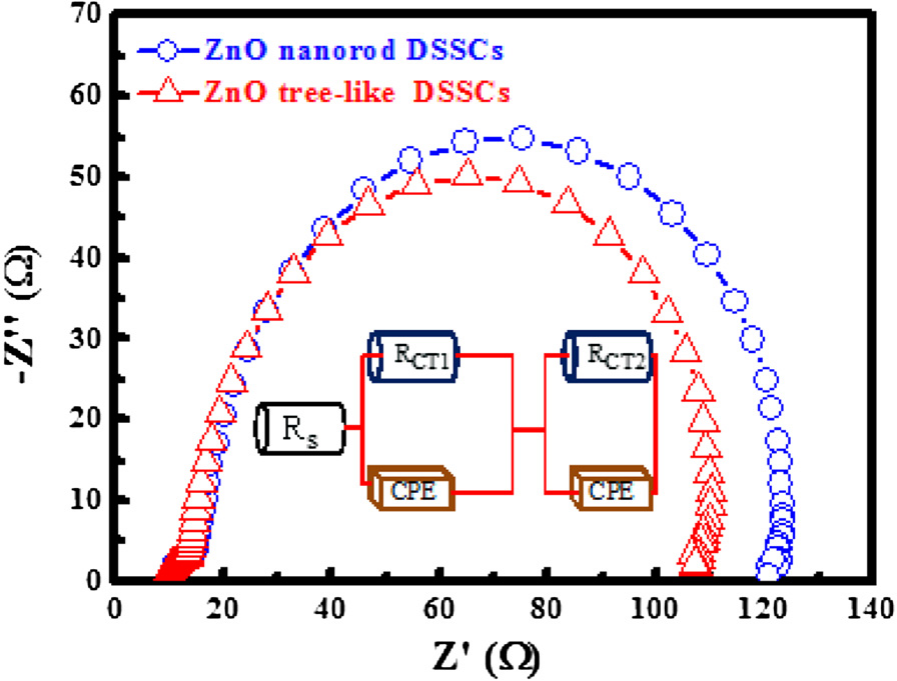
**Analysis of electrochemical impedance spectroscopy.** EIS of different ZnO nanostructure electrodes. Nyquist plots are used to measure under illumination (100 mA cm^−2^).

ZnO NRs and tree-like ZnO structures were obtained on an FTO substrate, and DSSCs were constructed, as shown in Figure 3. Figure 3a,b,c,d shows the FE-SEM images of the ZnO ‘NRs’ and ‘tree-like structures’ on the FTO substrate, respectively, indicating that the ZnO NRs are well-grown on the substrates with a distinctive, clear morphology. Both the lengths of the NRs and tree-like structures are in the range of 2 to 3 μm, as shown in Figure 3a,c. Figure 3a,b,c,d shows that the pillar-shaped tree-like structures form upright against the FTO substrate, whereas Figure 3a,c indicates that the NRs grow randomly on the FTO substrate. The eventual growth of tree-like ZnO structures or NRs was highly dependent on the preexisting textured seed layers on the FTO substrate. According to Greene et al., the factor causing the upright growth of ZnO NRs is the temperature during growth. In the present case, the growing temperature for the FTO substrate was set to be 90°C. Accordingly, the ZnO NRs grow on the FTO substrate, as shown in Figure 3c. To synthesize the branched structures of tree-like ZnO, a second set of AZO seeds containing the previously grown ZnO NRs were sputtered. The growth procedures at the same growth conditions were repeated. Figure 3a,b shows the tree-like ZnO with a branched structure. The dye loading at an approximate wavelength of 370 and 530 nm corresponds to the absorption edge of D-719 dye. Figure 4 shows the absorptions of solutions containing 0.01 mM dye, dyes detached from the tree-like ZnO structure films(red line), and dyes detached from the ZnO nanorod films (blue line). The solution was composed of 5 ml H_2_O and 0.1 mM NaOH. The films both had an area of 1 cm^2^. Figure 4 enables the calculation of the dye loadings and the light absorptions at 370 and 530 nm (the dye's absorption maximum) for both NRs and tree-like films. Compared to the upstanding ZnO NRs film, the tree-like film shows an improvement in both light harvesting and dye loading. The Nyquist plots of the impedance spectra are shown in Figure 5. To characterize the ZnO/dye/electrolyte interface characteristics, the DSSCs were at *V*_oc_ under AM 1.5 illumination by EIS measurement. The Nyquist plots (Figure 5) show a large semicircle at low frequencies and a small semicircle at high frequencies. As shown in Figure 5, they were fitted with an equivalent circuit alike to those reported in the literature. The equivalent circuit comprises *R*_s_ (ohmic resistance), *R*_ct1_ (the Pt counter electrode), and *R*_ct2_ (ZnO/dye/electrolyte interfaces):

(1) $$\tau_{\mathrm{eff}}={\left(2\pi f_{\text{min}}\right)}^{-1}$$

where τ_eff_ is the electron efficacious lifetime and *f*_min_ is the frequency corresponding to the imaginary part minimum. *R*_ct_ and *τ*_eff_ are reported in Table 1. Here, it is shown that the interface area increases and *R*_ct2_ decreases for tree-like nanostructures. The electrochemical parameters were evaluated by fitting the experimental data with the equivalent circuit, as summarized in Table 1. The *R*_CT2_ value for the photoelectrode containing a tree-like structure (95.8 Ω) is lower than that of the photoelectrode containing a nanorod structure (109.2 Ω), whereas the *R*_CT1_ value is almost the same. One possible cause for low-load transport resistance might be that axial charge transport in tree-like ZnO structures effectively obstructs the recombination progress with iodine redox carriers [8].

**Table 1:** Electrochemical and photovoltaic parameters of DSSCs

**Sample**	***V***_**oc **_**(V)**	***J***_**sc **_**(mA/cm**^**2**^**)**	**FF**	***R***_**ct2 **_**(Ω)**	***τ***_**eff **_**(ms)**	**Eff (%)**
NRs	0.661	0.699	0.397	109.2	3.23	0.203
Tree-like	0.680	0.784	0.413	95.8	3.91	0.231

Regarding branch-free rods, less accumulation on the electrode layer leads to poor electrolyte filling, improving the recombination pathway and raising the charge transport resistance. The surface charge density and trap level of the ZnO layer also play an important role in deciding the charge transport resistance by depleting the space charge layer. The drift transport that occurs when a 1D photoanode contacts electrolyte redox carriers is detrimental to efficacious charge transport. This requires further discussion [22,12]. EIS measurement was used to obtain the Bode plots of the lifetimes displayed in Table 1. This table shows that the tree-like ZnO structure DSSCs exhibit a longer electron lifetime (*τ*_eff_ = 3.91 ms) than that of the NRs DSSCs (*τ*_eff_ = 3.28 ms). The longer lifetime implies lower recombination rate and increased electron-collection efficiency, and thus the parameter can be related to the improvement in cell efficiency.

**Figure 6:**
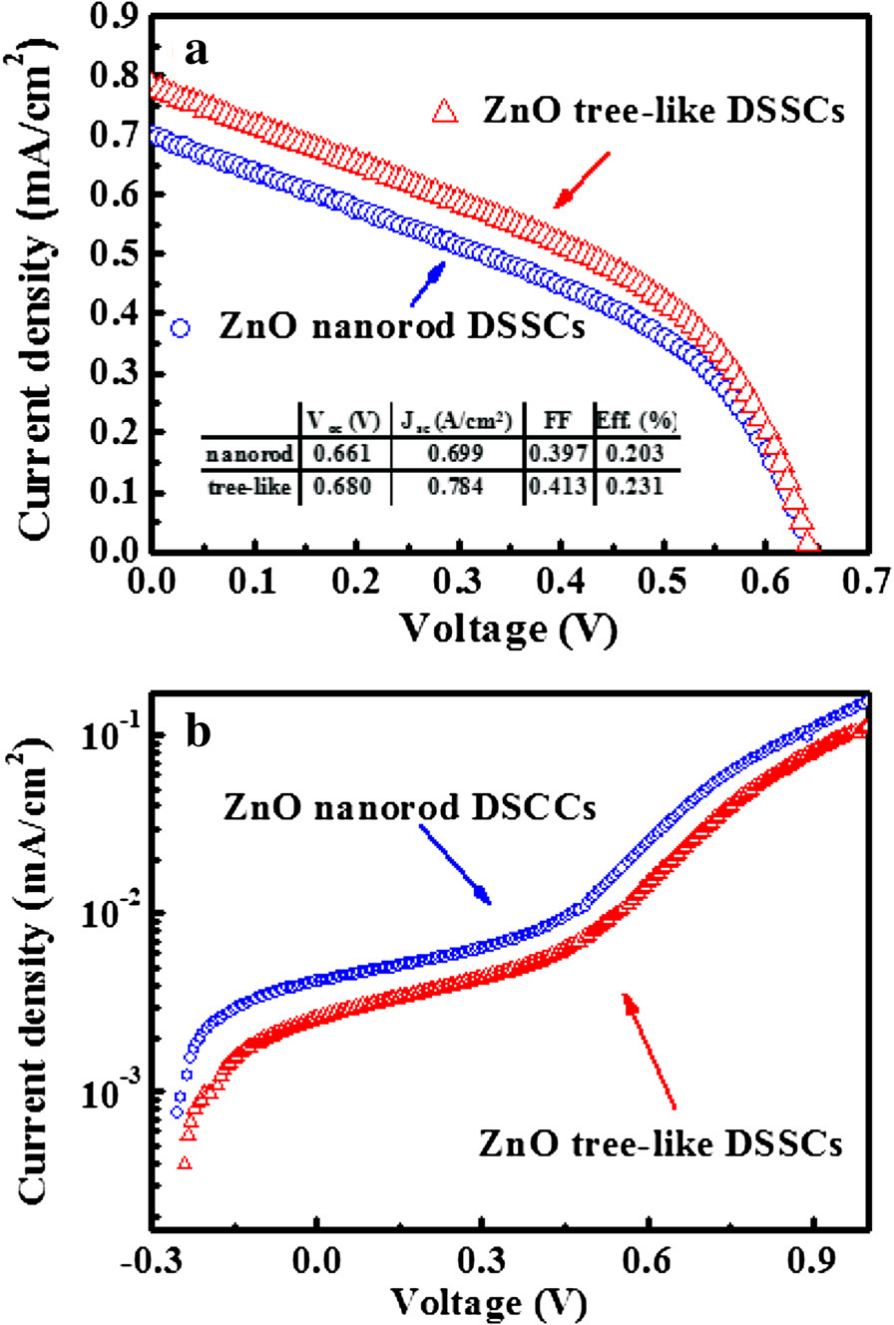
**Current-voltage characteristics**. *J*-*V* measurements under **(a)** light illumination (100 mA cm^−2^) and **(b)** dark illumination.

Figure 6a shows the *J*-*V* curve for the DSSCs composed of tree-like structures and NRs. The DSSC made of NRs yields power conversion efficiency (*η*) of 0.20%. The DSSC derived from tree-like nanostructures demonstrates an increased power conversion efficiency of 0.23%, and the enhancement in power conversion reaches 15%. As shown in Figure 6a, short circuit current (*J*_sc_), open circuit voltage (*V*_oc_), and fill factor (FF) are all substantially increased in the tree-like structures compared to that of the NRs. These factors all contribute to increasing power conversion efficiency. The increased *J*_sc_ in tree-like ZnO nanostructure DSSCs can be attributed to the large internal surface area for dye anchoring and the effective conduction pathway provided by the highly interconnected network of the branched structure. Additional random multiple scattering of light within the network also possibly leads to photon localization, thereby increases the probability of light harvesting.

The *V*_oc_ for the tree-like ZnO nanostructures also increased compared to that of the ZnO nanorods. This higher *V*_oc_ is attributed to a reduction in recombination losses at ZnO/dye interfaces. The high *V*_oc_ for the tree-like ZnO nanostructure DSSCs can be solved with the diode equation [23]:

(2) $$V_{\mathrm{oc}}=\left(\frac{\mathrm{KT}}{\mathrm{nq}}\right)\ln\left(\frac{I_{\text{max}}}{I_{o}}\right)$$

where the *I*_max_ and *I*_0_ are the maximum current density and dark current density, respectively, in Equation 2. This equation predicts that the suppression of the dark current density (*I*_0_) results in a higher *V*_oc_, and the enhancement of *J*_sc_ is almost 12%. Accordingly, Figure 6b shows that the dark current density of DSSC with ZnO tree-like nanostructure was lower than that with ZnO nanorod. The dark current density supplies qualitative information on dye coverage on the photoelectrode surface [24]. The lower dark current density in the tree-like ZnO nanostructure photoelectrode is caused by efficient dye coverage on the surface of the ZnO branches, as well as proper electrolyte penetration. These factors result in low recombination damages at ZnO/dye interfaces. Furthermore, the *V*_oc_ increase in tree-like nanostructure DSSCs can be explained in two ways: (1) Higher dye loading fosters more charge injection from the dye sensitizer to the conduction band of ZnO. The result is an upward shift in the ZnO quasi-Fermi level, thus enhancing the potential difference between ZnO and the redox species. (2) Sufficient electrolyte pore filling in vertically branched structures leads to efficient hole scavenging at ZnO/dye interfaces, lowering the locus of recombination [25].

Although the power conversion efficiency of the present work is lower than the highest value reported in the literature [6], our principal concern is on whether the tree-like nanostructure can improve on the conversion efficiency of a DSSC composed of nanorods. This study determined that a tree-like ZnO nanostructure synthesized through effortless and gentle reaction conditions is highly efficient and economically viable as a photoelectrode for DSSCs. Further work will improve the cell configuration and conversion efficiency.

## Conclusions

This study prepared tree-like ZnO structures and ZnO nanorods for use as photoanodes in DSSCs. DSSCs composed of tree-like ZnO nanostructures were found to show greater photovoltaic performance than DSSCs containing nanorods. Comparatively, tree-like ZnO structures exhibit a larger internal surface area for efficient dye loading and light harvesting, a greater available pore volume, reduced charge recombination, and improved interconnectivity for faster electron transport than ZnO nanorods. These improvements yield a 15% enhancement in power conversion.

## Competing interests

The authors declare that they have no competing interests.

## Authors’ contributions

SYK and FIL supervised the research and revised the manuscript. JFY designed and carried out the experiment and statistical analysis and participated in drafting the manuscript. All authors read and approved the manuscript.
